# The Training Characteristics of the World's Most Successful Female Cross-Country Skier

**DOI:** 10.3389/fphys.2017.01069

**Published:** 2017-12-18

**Authors:** Guro S. Solli, Espen Tønnessen, Øyvind Sandbakk

**Affiliations:** ^1^Department of Sports Science and Physical Education, Nord University, Bodø, Norway; ^2^The Norwegian Olympic Federation, Oslo, Norway; ^3^Department of Neuromedicine and Movement Science, Centre for Elite Sports Research, Norwegian University of Science and Technology, Trondheim, Norway

**Keywords:** altitude training, endurance training, high-intensity training, performance, periodization, speed training, strength training, tapering

## Abstract

The main aim of this study was to investigate the training characteristics of the most successful female cross-country skier ever during the best period of her career. The participant won six gold medals at the Olympic Games, 18 gold medals at the World Championship, and 110 World Cup victories. Day-to-day training diary data, interviews, and physiological tests were analyzed. Training data was systemized by training form (endurance, strength, and speed), intensity [low- (LIT), moderate- (MIT), and high-intensity training (HIT)], and mode (running, cycling, and skiing/roller skiing), followed by a division into different periodization phases. Specific sessions utilized in the various periodization periods and the day-to-day periodization of training, in connection with altitude camps and tapering toward major championships, were also analyzed. Following a 12-year nonlinear increase in training load, the annual training volume during the five consecutive successful years stabilized at 937 ± 25 h, distributed across 543 ± 9 sessions. During these 5 years, total training time was distributed as 90.6% endurance-, 8.0% strength-, and 1.4% speed-training, with endurance-training time consisting of 92.3 ± 0.3% LIT, 2.9 ± 0.5% MIT, and 4.8 ± 0.5% HIT. Total LIT-time consisted of 21% warm-up, 14% sessions <90 min, and 65% long-duration sessions >90 min. While the total number of LIT sessions remained stable across phases (32 sessions), total LIT-time was reduced from GP (76 h/month) to SP (68 h/month) and CP (55 h/month). MIT-time decreased from GP (2.8 h/month) to SP (2.2 h/month) and CP (1 h/month). HIT-time increased from GP (2.8 h/month) to SP (3.2 h/month) and CP (4.7 h/month). Altitude training accounted for 18–25% of annual training volume and performed across relatively short training camps (≤16 days) with a clear reduction of HIT training, but increased total and LIT volume compared to sea-level training. Training before international championships included a 2-week increase in LIT and strength volume followed by a gradual reduction of training volume and increased HIT during the last week. This study provides unique data on the world's most successful female cross-country skier's long-term training process, including novel information about the distribution of and interplay between sessions of different forms, intensities, and exercise modes throughout the annual season.

## Introduction

Cross-country (XC) skiers optimize their training to perform in competitions ranging from multiple 3-min sprint races to prolonged endurance races lasting up to 2 h. These competitions are performed across varying terrain while changing between the different sub-techniques in classic and skating (Sandbakk and Holmberg, 2017). Although the average aerobic energy contribution is 70–75% in sprint races and 85–95% for longer distances, the race format is interval-based, with increased effort in uphill terrain and lower intensities downhill (Norman et al., 1989; Sandbakk et al., 2011a; Sandbakk and Holmberg, 2017). Furthermore, the majority of competitions involve mass-starts in which sprint ability is critical in determining the final result. Accordingly, high aerobic capacity is of crucial importance in XC skiing, as reflected by world-class XC skiers' high *V*O_2max_ values (>80 and ~70 mL·kg^−1^·min^−1^ for men and women, respectively) (Saltin and Astrand, 1967; Ingjer, 1991; Sandbakk et al., 2011b, 2016; Tonnessen et al., 2015a). However, XC skiers also need the ability to rapidly elevate their peak oxygen uptake, utilize a high fraction of their *V*O_2max_ in all of the sub-techniques, and have well-developed skiing efficiency and anaerobic capacity (Sandbakk and Holmberg, 2017). Unique for XC skiing, both training and competitions involve large fluctuations in speed, work rate, and metabolic intensity, in addition to a varying load on the upper and lower body with the different training modes and sub-techniques utilized. Hence, the training of XC skiers is made up of a sophisticated puzzle of training sessions of different forms, intensities, and exercise modes that has not yet been examined in detail.

World-class XC skiers have previously reported 800–950 annual training hours (Tonnessen et al., 2014; Sandbakk et al., 2016). Of the total annual training volume, >90% of elite XC skiers' has been reported to be endurance training, with the remaining ~10% performed as strength or speed work (Sandbakk et al., 2011b, 2016; Tonnessen et al., 2014). These studies showed a pattern of endurance training time distributed as 88–91% low-intensity training (LIT, < VT_1_ aerobic threshold), 3–7% moderate-intensity (MIT, VT_1_ < >VT_2_ anaerobic threshold), and 5–8% high-intensity training (HIT, >VT_2_). As also shown in other endurance sports (Stoggl and Sperlich, 2015), a pyramidal training pattern is often found during the preparation period, whereas more polarized training is done in the competition phase.

Approximately 60% of the total training time is performed during the general preparation period between May and October. This period typically includes high volumes of LIT and 50–60% of the endurance-training conducted as sport-specific exercise (e.g., roller skiing and skiing), with the remainder mainly performed as running (Tonnessen et al., 2014; Sandbakk et al., 2016). The remaining 40% of annual training is performed during the specific preparation and competition phase, with decreased total volume, increased amount of HIT (including 30–40 competitions) and higher amount of sport-specific activity forms (Sandbakk and Holmberg, 2017). While the intensity distribution and use of activity forms during different phases of the year are well covered by several retrospective studies (Sandbakk et al., 2011b, 2016; Tonnessen et al., 2014), the design of specific sessions within the different zones and modes are not well illustrated in the current literature.

In terms of periodization, most XC skiers use a traditional model, alternating between high- and low-volume weeks while keeping the number of MIT and HIT sessions relatively stable (two to three sessions per week; Tonnessen et al., 2014). However, some athletes organize the training in blocks with increased focus on developing specific capacities over shorter periods (Sandbakk and Holmberg, 2017). Altitude training represents a significant portion of world-class XC skier training and is an important piece of the periodization puzzle (Sandbakk et al., 2016). The main aim of these altitude training camps (living at ~1,800–2,000 m above sea-level and training at 1,000–3,000 m) is mainly to positively stimulate hematological parameters, and thereby improve performance during the subsequent training and/or competition period (Millet et al., 2010). However, in XC skiing, altitude training also provides an opportunity for many hours of skiing on snow throughout the dry-land training period (Sandbakk and Holmberg, 2017). Although training at altitude has been used by endurance athletes for several decades, accurate descriptions of the micro periodization of successful athletes is lacking in the literature.

To ensure peak performance, a typical tapering approach has been to perform 2–4 weeks of overload training, followed by 1–3 weeks with decreased load (Hellard et al., 2013). Bosquet et al. (2007) reported that performance improvements are highly sensitive to reductions in training volume and that the optimal range of volume reduction is 41–60%, without substantial decreases in training frequency, compared to the pre-tapering training. However, a recent study (Tonnessen et al., 2014) observed that gold medal winning athletes in XC skiing and biathlon used a more modest and progressive reduction in training volume, with a relatively small reduction during the last weeks prior to gold-medal performance. The authors speculate that this progressive tapering strategy could be ideal in sports with a dense competition schedule, as is the case in XC skiing. However, the study reports large individual differences in tapering behavior and does not consider the specific sessions utilized during the final phases of the taper. Thus, tapering behavior in world-class endurance athletes is lacking, particularly in athletes who have attained repeated success in major championships.

The training of world-class XC skiers involves manipulation of variables such as different training forms (endurance, strength, and speed), exercise modes (loading of the upper and/or lower body), session organization (continuous or interval), and varying terrain, making it more complex than many other endurance sports. This makes it particularly challenging to investigate at a group level. Case studies allow us to investigate every piece of the training in detail and expand our understanding of champion performance development. Earlier case studies of high-level endurance athletes have focused on physiological test data (Jones, 1998, 2006; Bell et al., 2017) or short-term training studies (Stellingwerf, 2012; Mujika, 2014; Manunzio et al., 2016; Ronnestad et al., 2017). Only a minority of studies consist of longitudinal training data spanning several years, and the majority of these focus on male subjects (Ingham et al., 2012; Tjelta, 2013; Bourgois et al., 2014; Tjelta et al., 2014; Pinot and Grappe, 2015).

Therefore, the primary aim of this study was to investigate the training characteristics of the most successful female XC skier ever during the best period of her career, including the day-to-day periodization of her training in connection with altitude camps and tapering toward major international competitions. In order to interpret these findings in the perspective of her long-term development process, the secondary aim was to characterize her longitudinal training patterns over 17 years.

## Methods

### Participant

The participant (born in 1980) is the most successful female competitor of all time in the winter Olympics and Nordic skiing World Championships. This includes six Olympic gold medals (four individual, two team), 18 gold medals from the FIS World Ski Championship (12 individual, six team), 110 individual FIS World-Cup victories, and four wins of the overall FIS World Cup (FIS, 2017). The study was evaluated by the regional ethics committee of mid-Norway, and approved by the Norwegian Social Science Data Services (NSD). Written informed consent was obtained from the participant for the publication of this study, which was performed according to the Helsinki declarations.

### Overall design

To give both comprehensive understanding of and detailed insight into the athlete's training, the study was divided into two parts (1) investigations of the participant's longitudinal training, performance, and test data spanning 17 years from the age of 20 to 37 years old (2000–2017); and (2) detailed investigations of the training during five consecutive successful seasons from the age of 30 to 35 years old (2010–2015), including four international championships with nine individual gold medals.

### Performance data

Performance data for each year was calculated using both race results and the International Ski Federation's (FIS) ranking points from all individual distance and sprint competitions, including World Cup competitions, the Olympic Games, and the FIS World championships (FIS, 2017).

### Physiological testing

The participant underwent regular *V*O_2max_ and lactate profile testing (test results presented in Table 1). No physiological tests were performed during the competition period (CP), and the presented results therefore represent tests from May or June (the start of the general preparation period; GP) and October or November (the start of the specific preparation period: SP). All physiological testing during the period was conducted at the Norwegian Olympic Sports Centre, primarily supervised by the same exercise physiologist. The apparatus and testing procedures used during the lactate profile and *V*O_2max_ test are previously described (Ingjer, 1991; Tonnessen et al., 2015a). Anaerobic threshold (AT) was determined during treadmill running at 10.5% incline using a graded protocol, including 4–6 periods of 5-min stages with stepwise 1-km/h increases in workload (Enoksen et al., 2011). The same treadmill (Woodway Gmbh, 124 Weil am Rhein, Germany) was used at all tests and lactate concentration was measured from the fingertip by an YSI 1500 sport lactate analyzer (YSI, Ohio, USA) directly after completion of each stage. *V*O_2_ was recorded between the third and fourth minute at each stage using an Oxycon Pro (Jaeger-Toennis, Wurtzburg, Germany) metabolic test system. AT was determined at the workload corresponding to 1.5 mmol/1 higher lactate concentration than the baseline value (averaged over the two first measurements). Total, lean, and fat mass were analyzed for the legs, trunk, arms, and head using dual-energy X-ray absorptiometry (DXA) (Encore 2007, Version 11.4, General Electric Medical Systems, Madison, WI, USA), and presented in absolute values (Table 1).

**Table 1:** Physiological characteristics of the world's most successful female cross-country skier during the successful period from 2010 to 2015.

	**2010**		**2011**		**2012**		**2013**		**2014**		**Mean ± SD**
	**GP1**	**GP2**	**GP1**	**GP2**	**GP1**	**GP2**	**GP1**	**GP2**	**GP1**	**GP2**	
Age (year)	30	30	31	31	32	32	33	33	34	34	32.0 ± 1.5
Body height (cm)	167	167	167	167	167	167	167	167	167	167	167.0 ± 0.0
Body mass (kg)	65.4	64.6	64.9	64.2	65.7	64.6	65.2	65.2	64.1	64.0	64.8 ± 0.6
Body mass index (kg·m^−2^)	23.5	23.2	23.3	23.0	23.6	23.2	23.4	23.4	23.0	22.9	23.2 ± 0.2
Lean body mass (kg)	–	–	–	–	–	54.9	54.5	–	54.6	55.0	54.8 ± 0.2
Lean upper body mass (kg)	–	–	–	–	–	35.0	34.1	–	34.0	34.5	34.4 ± 0.5
Lean lower body mass (kg)	–	–	–	–	–	18.3	17.4	–	17.5	17.6	17.7 ± 0.4
Total body fat (%)	–	–	–	–	–	14.8	15.2	–	14.2	12.8	14.3 ± 1.1
*V*O_2max_ (L·min^−1^)^*^	4.23	4.49	4.31	4.39	4.47	4.52	4.33	4.37	4.42	–	4.39 ± 0.1
*V*O_2max_ (ml·kg^−1^·min^−1^)^*^	64.7	69.5	66.4	68.4	68.0	70.0	66.4	66.7	69.0	–	67.7 ± 1.7
*V*O_2@AT_ (ml·kg^−1^·min^−1^)^*^	58.9	61.1	57.0	59.2	60.7	63.6	58.7	61.0	59.4	60.8	60.0 ± 1.8
V_@AT_ (km/h)^*^	9.8	10.9	10.3	10.7	10.8	10.9	10.6	10.8	10.7	11.2	10.7 ± 0.4

VO_2max_, maximal oxygen uptake; AT, estimated anaerobic threshold; VO_2@AT_ oxygen uptake at the anaerobic threshold (running); V@AT, velocity at the anaerobic threshold; GP1, general preparation period one; GP2, general preparation period two;

* *, gradient of treadmill 10.5%*.

### Training monitoring

The participant recorded her day-to-day training in digital diaries designed by the Norwegian Ski Association and the Norwegian Olympic Federation. The training recorded for each session included total training time distributed across training form (endurance, strength, and sprint), activity form (skiing, roller-skiing, running, cycling, etc.), and intensity zone. Specific comments regarding session details were also recorded.

To register the endurance-training intensity, the five-zone intensity scale developed by the Norwegian Olympic Federation was used, which has been reported to provide a valid and accurate measurement of the duration and intensity of training by XC skiers (Sylta et al., 2014a). However, since these zone boundaries do not clearly correspond with underlying physiological events (Boulay et al., 1997), we used a three zone scale based on the ventilatory changes corresponding to the first- and second-lactate turning point (Boulay et al., 1997; Seiler and Kjerland, 2006). LIT refers to a training intensity below the first lactate threshold (LT^1^) (<2 mM blood lactate, 60–82% of maximal heart rate; HR_max_). Moderate-intensity training (MIT) refers to an intensity between LT^1^ and LT^2^ (2–4 mM blood lactate, 82–87% of HR_max_). High-intensity training (HIT) refers to an intensity above LT^2^ (>4 mM blood lactate, >87% of HR_max_) (Seiler and Kjerland, 2006). Standardized intensity scales do not take into account the individual or activity-specific variation, such as the tendency for maximal steady-state concentrations of blood lactate tending to be higher in activities activating less muscle mass (Beneke and von Duvillard, 1996; Beneke et al., 2001). Hence, the participant in this study tailored her intensity zones in accordance with both test results and her own experience. Her self-reported intensity zones are presented in Table 2.

**Table 2:** Self-reported intensity zones presented as maximal, minimal and most commonly used (target) heart rates in the specific training zones, as well as the average rating of perceived exertion across the different categories of endurance sessions for the world's most successful female cross-country skier.

**Intensity zones**	**HR zones**			**RPE**	**Session categories**
	**Min** **Beat · min^−1^ (% HR_max_)**	**Target** **Beat · min^−1^ (% HR_max_)**	**Max** **Beat · min^−1^ (% HR_max_)**		
LIT^*^	115 (67)	115–130 (67–75)	149 (86)	11	Warm up and cool down^**^ Short-duration session < 50 min Medium-duration session [50–90 min> Long-duration session [90–150 min > Very long-duration session ≥ 150 min
MIT	150 (87)	155–160 (89–92)	160 (92)	15	Continuous training Intervals with periods from 10 to 15 min Intervals with periods from 6 to 10 min
HIT	161 (93)	161–170 (93–98)	173 (100)	19	Continuous training^#^ Intervals with periods from 4 to 7 min Intervals with periods < 4 min^##^

*LIT, low-intensity training; MIT, moderate-intensity training; HIT, high-intensity training; RPE, Rating of perceived exertion (BORG scale, 6–20)*.

* *When sprints were integrated into LIT sessions, sprint time (including 1-2 min recovery after each sprint) was subtracted from the overall duration of the session. The remaining time was categorized as LIT*.

** *The category includes LIT performed as warm up or cool down in connection with MIT, HIT and strength sessions*.

# *Including distance competitions*.

## *Including sprint competitions*.

### Registration and systematization of training data

To register training time, the participant used a combination of the session-goal approach and time in training zone often called a *modified session-goal approach*, described in detail by (Sylta et al., 2014b). The participant registered endurance training by allocating the time of the different parts of the sessions (e.g., warm-up, intervals, and cool-down) into intensity zones based on actual HR registration supported by external load, lactate measurements, and self-perceived exertion. For MIT and HIT sessions performed as intervals, the time in the MIT/HIT zone was registered from the beginning of the first interval to the end of the last interval, including recovery periods. Strength and speed training was registered from the start to the finish of the specific strength/speed/jump part of the session, including recovery periods. When speed training was integrated into LIT sessions, 2 min per sprint was registered as speed training.

All data from training diaries were systematically analyzed session-by-session by researchers from the current research group. Total training time and frequency of sessions were distributed in line with “the training distribution method” previously described (Tonnessen et al., 2014). All endurance sessions were categorized based on duration and/or design, as presented in Table 2.

### Periodization phases

General training data are either presented as annual training characteristics or divided into different periodization phases, as presented in Table 3. The day-to-day periodization of training before, during, and after altitude camps is quantified based on the final 2 weeks prior to altitude training, the first 2 weeks of altitude training, and the 2 weeks after the altitude camp in October. The training during and after the second week of the altitude camp in 2012 was excluded from the analysis because of illness. Tapering characteristics are quantified based on the six final weeks of training prior to the FIS World Championships in 2011, 2013, and 2015 and the Olympic Games in 2014.

**Table 3:** The division of periodization phases across the annual training cycle, including altitude- and peaking phases^*^.

**Phase**	**Period in annual training cycle**	**Duration (days)**
General preparation Period (GP)	May–October	184
General preparation Period 1 (GP1)	May–July	92
General preparation Period 2 (GP2)	August–October	92
*Pre-altitude phase*	*Day 14-1 before altitude camp*	*14*
*Altitude phase*	*Day 1-14 of the altitude camp*	*14*
*After-altitude phase*	*Day 1-14 after altitude camp*	*14*
Specific preparation period (SP)	November–December	61
Competition period (CP)	January–March	90
*Pre-peaking Phase 1*	*Day 42-29 before first championship event*	*14*
*Pre-peaking Phase 2*	*Day 28-15 before first championship event*	*14*
*Peaking Phase*	*Day 14-1 before first championship event*	*14*

* *April was defined as regeneration period and was not included in any of the other periods. However, training time in April is included in the calculation of the total annual training*.

### Interviews

To track missing data, ensure compliance with the training diary commentaries, and verify the training intensity of different training sessions, two structured and one semi-structured interview with the participant were conducted during the data-analysis phase of this study.

### Missing data

Training information was lacking for March and April of the 2010/2011 season. Data for these months was calculated based on the years in which data was completely documented and modified based on training plans and an interview with the athlete. Sessions where information about session design was lacking (10% of MIT sessions and 3% of the HIT sessions) were only used in the time-in zone analyses.

### Statistical analyses

All data from the 2010–2015 period is presented as mean ± standard deviation (SD) of the five years. To calculate the monthly and weekly distribution of training, total training was divided by duration (days) of the specific phase and multiplied by 30.4 to determine monthly time/frequency (**Figures 2, 3A–D, 4A–C**) or by seven to determine the weekly time and frequency (Table 4 and **Figures 5A,B**). All statistical analyses were carried out in Microsoft Office Excel 2013 (Microsoft, Redmond, WA, USA).

**Table 4:** Weekly training distribution (mean ± SD) across the diffrent periodization phases including the different phases of the altitude camp performed in October and the 6 weeks prior to international championships during the five successfull years from 2010–2015 for the world's most successful female cross-country skier.

	**GP1**	**GP2**				**SP**	**CP**			
		**Overall**	**Pre altitude**	**During altitude**	**After altitude**		**Overall**	**Pre-peaking Phase 1**	**Pre-peaking Phase 2**	**Peaking phase**
**TOTAL TRAINING**										
Hours	20.9 ± 1.3	21.7 ± 0.6	19.9 ± 1.0	27.6 ± 1.6	18.3 ± 1.5	18.4 ± 0.5	14.7 ± 0.8	15.8 ± 2.2	19.7 ± 2.7	16.2 ± 0.5
Sessions	10.8 ± 0.3	11.1 ± 0.2	10.9 ± 0.4	11.9 ± 0.6	10.3 ± 0.9	11.7 ± 0.5	10.5 ± 0.6	10.8 ± 1.0	11.3 ± 1.5	11.9 ± 0.3
**TRAINING FORMS**										
Endurance (h)	18.3 ± 0.6	19.4 ± 0.6	16.9 ± 0.4	26.1 ± 1.0	16.2 ± 1.7	16.9 ± 0.4	14.0 ± 0.7	15.2 ± 2.0	17.6 ± 2.1	15.3 ± 0.7
Strength (h)	2.2 ± 0.6	1.9 ± 0.7	2.6 ± 0.6	1.4 ± 0.8	1.8 ± 0.7	1.2 ± 0.3	0.6 ± 0.2	0.5 ± 0.0	1.9 ± 0.9	0.6 ± 0.5
Speed (h)	0.4 ± 0.1	0.4 ± 0.1	0.4 ± 0.2	0.2 ± 0.1	0.3 ± 0.2	0.2 ± 0.0	0.1 ± 0.0	0.1 ± 0.1	0.2 ± 0.1	0.3 ± 0.1
**EXERCISE MODE**										
Specific (h)	9.8 ± 0.5	10.0 ± 0.5	7.1 ± 2.3	19.2 ± 4.8	9.2 ± 1.1	13.3 ± 1.2	12.0 ± 0.8	13.8 ± 2.0	15.3 ± 2.1	13.6 ± 0.7
Unspecific (h)	8.8 ± 0.6	9.7 ± 0.4	10.2 ± 2.9	7.1 ± 2.9	7.3 ± 0.9	3.8 ± 0.9	2.1 ± 0.3	1.5 ± 0.6	2.5 ± 0.6	2.0 ± 0.2
SPE/UNSPE (%)	53/47	51/49	41/59	73/27	56/44	78/22	85/15	90/10	86/14	87/13
**INTENSITY DISTRIBUTION**										
LIT (h)	17.2 ± 0.5	17.9 ± 0.6	15.4 ± 0.5	24.7 ± 1.1	14.8 ± 1.8	15.7 ± 0.4	12.7 ± 0.7	14.0 ± 2.1	16.2 ± 0.9	14.0 ± 1.6
MIT (h)	0.6 ± 0.2	0.7 ± 0.1	0.4 ± 0.4	1.2 ± 0.3	0.7 ± 0.2	0.5 ± 0.1	0.2 ± 0.0	0.3 ± 0.1	0.4 ± 0.3	0.3 ± 0.2
HIT (h)	0.5 ± 0.1	0.8 ± 0.1	1.1 ± 0.2	0.2 ± 0.2	0.7 ± 0.2	0.7 ± 0.1	1.1 ± 0.1	0.9 ± 0.2	1.0 ± 0.1	1.0 ± 0.1
LIT/MIT/HIT (%)	94/3/3	92/4/4	91/2/7	94/5/1	92/4/4	93/3/4	91/1/8	92/2/6	92/2/6	91/2/7
**INTENSITY DISTRIBUTION**										
LIT (sessions)	7.1 ± 0.3	6.9 ± 0.3	6.1 ± 1.2	8.6 ± 0.8	6.6 ± 0.8	7.8 ± 0.5	7.3 ± 0.5	7.1 ± 0.9	7.1 ± 1.0	8.3 ± 0.5
MIT (sessions)	0.8 ± 0.2	0.9 ± 0.1	0.7 ± 0.8	1.6 ± 0.3	0.9 ± 0.3	0.8 ± 0.2	0.5 ± 0.1	0.8 ± 0.5	0.5 ± 0.4	0.6 ± 0.3
HIT (sessions)	0.9 ± 0.2	1.6 ± 0.1	2.2 ± 0.3	0.3 ± 0.3	1.3 ± 0.3	1.9 ± 0.3	2.1 ± 0.2	2.3 ± 0.3	2.0 ± 0.0	2.3 ± 0.3
LIT/MIT/HIT (%)	80/9/11	74/9/17	68/8/24	82/16/2	76/10/14	74/8/18	74/5/21	71/7/22	74/5/21	74/6/20
**CATEGORIZATION OF LIT**										
<50 min (sessions)	0.2 ± 0.2	0.1 ± 0.1	0.0 ± 0.0	0.0 ± 0.0	0.0 ± 0.0	1.9 ± 0.7	2.8 ± 0.5	2.1 ± 0.5	1.1 ± 0.8	3.0 ± 0.4
50–90 min (sessions)	1.0 ± 0.2	1.1 ± 0.3	1.1 ± 1.0	0.5 ± 0.4	1.5 ± 1.1	1.7 ± 0.6	1.3 ± 0.4	1.4 ± 0.8	1.3 ± 0.5	2.4 ± 1.7
90–150 min (sessions)	4.4 ± 0.5	4.0 ± 0.4	4.7 ± 0.8	3.9 ± 0.6	4.8 ± 1.0	3.2 ± 0.2	2.4 ± 0.4	3.0 ± 0.7	3.1 ± 0.8	2.4 ± 0.9
≥150 min (sessions)	1.6 ± 0.5	1.8 ± 0.1	0.5 ± 0.4	4.5 ± 0.7	0.5 ± 0.4	1.0 ± 0.4	0.7 ± 0.2	0.9 ± 0.8	1.8 ± 0.3	1.0 ± 0.7
**AVG. SESSION DURATION**										
LIT (h)	2.0 ± 0.1	2.1 ± 0.1	1.8 ± 0.1	2.6 ± 0.1	1.8 ± 0.1	1.5 ± 0.1	1.3 ± 0.1	1.4 ± 0.1	1.7 ± 0.1	1.3 ± 0.2
MIT (h)	0.8 ± 0.1	0.8 ± 0.1	0.7 ± 0.1	0.8 ± 0.1	0.7 ± 0.0	0.7 ± 0.1	0.5 ± 0.1	0.2 ± 0.1	0.4 ± 0.3	0.3 ± 0.2
HIT (h)	0.5 ± 0.0	0.5 ± 0.0	0.5 ± 0.1	0.7 ± 0.0	0.5 ± 0.0	0.4 ± 0.0	0.5 ± 0.1	0.4 ± 0.1	0.5 ± 0.1	0.5 ± 0.0
**COMPETITIONS**										
Hours	0.1 ± 0.1	0.1 ± 0.1	0.0 ± 0.0	0.0 ± 0.0	0.0 ± 0.0	0.4 ± 0.1	0.7 ± 0.1	0.6 ± 0.3	0.5 ± 0.3	0.3 ± 0.1
Number	0.2 ± 0.1	0.3 ± 0.2	0.0 ± 0.0	0.0 ± 0.0	0.0 ± 0.0	1.1 ± 0.2	1.5 ± 0.2	1.6 ± 0.5	1.0 ± 0.6	0.9 ± 0.3

*GP1, general preparation period 1; GP2, General preparation period 2; SP, Specific preparation period; CP, Competitions phase; SPE, Specific exercise mode; UNSPE, Unspecific exercise mode; LIT, Low intensity training; MIT, moderate intensity training; HIT, High intensity training*.

## Results

### Longitudinal training characteristics

**Figure 1:**
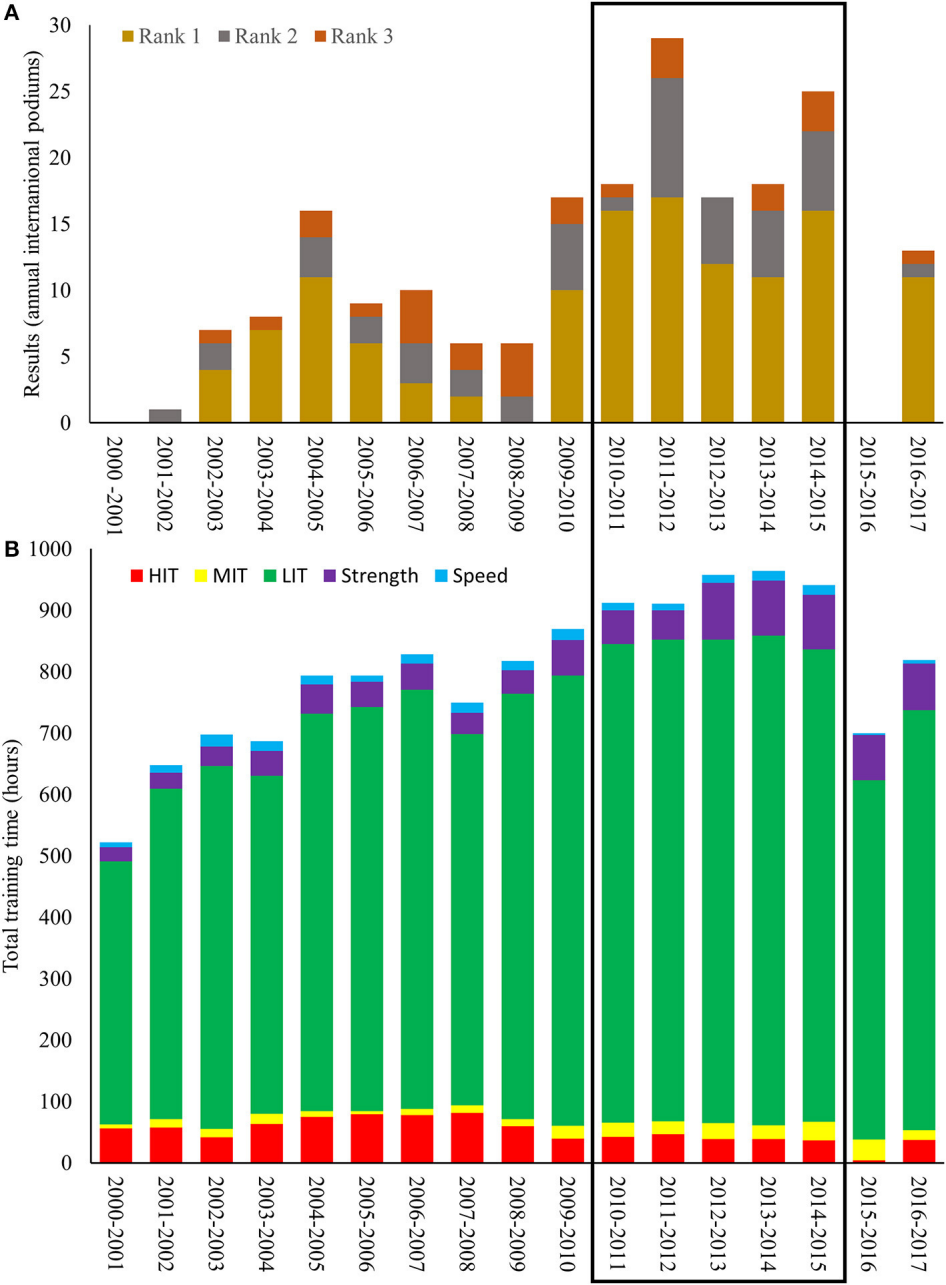
**(A,B)** Annual top three performances **(A)** in international competitions and annual training characteristics **(B)** distributed into endurance (low-, moderate-, and high-intensity), strength and speed training during a 17-year period for the world's most successful female cross-country skier.

In total, 8,105 training sessions were analyzed during the period from 2000 to 2017. These sessions comprised of 7,642 workouts and 463 XC skiing competitions. Performance and training data during all 17 years are presented in Figures 1A,B. Total annual training volume increased by 80% (from 522 to 940 h) from the age of 20–35 (2000–2015). This a yearly progression of 30 ± 53 h and an increase from ~10 to 18 weekly training hours. The relative distribution of endurance training into LIT/MIT/HIT was polarized, but to a lesser extent during the latter part of her career, i.e., ~88/2/10 during the first part of her senior career (20–27 years old) and ~92/3/5 during the latter part (28–35 years old). Subsequently, LIT volume increased from ~430 h (20 years old) to ~800 h (35 years old). The amount of MIT + HIT was ~60 h during both the early (20–23 years old) and latter (29–35 years old) stage of her career, but was markedly higher (~80 h) during a 5-year period from 23 to 28 caused by the use of extensive HIT blocks during the general preparation phase.

### Training characteristics of the five most successful seasons

During the five consecutive seasons, from May 2010 to April 2015, the participant achieved 107 individual podium places in international competitions, including Olympic Games, FIS World championships and FIS World Cup. This consisted of 63 individual World Cup victories, two gold medals from the 2014 Olympics and seven gold medals from the three World Championships. Annual ranking and FIS points in the abovementioned international races were 2.5 ± 0.8 (2.0 ± 0.8 in distance races and 3.4 ± 2 in sprint races) and 9.3 ± 3.0 (including distance races and sprint qualifications points), respectively.

Physiological tests (Table 1) showed an average *V*O_2max_ of 4.39 ± 0.09 (L·min^−1^) and 67.7 ± 1.7 (ml·kg^−1^·min^−1^) during the 5 years, with increased values from GP1 to GP2. *V*O_2_ at AT was approximately 89% of *V*O_2max_.

**Figure 2:**
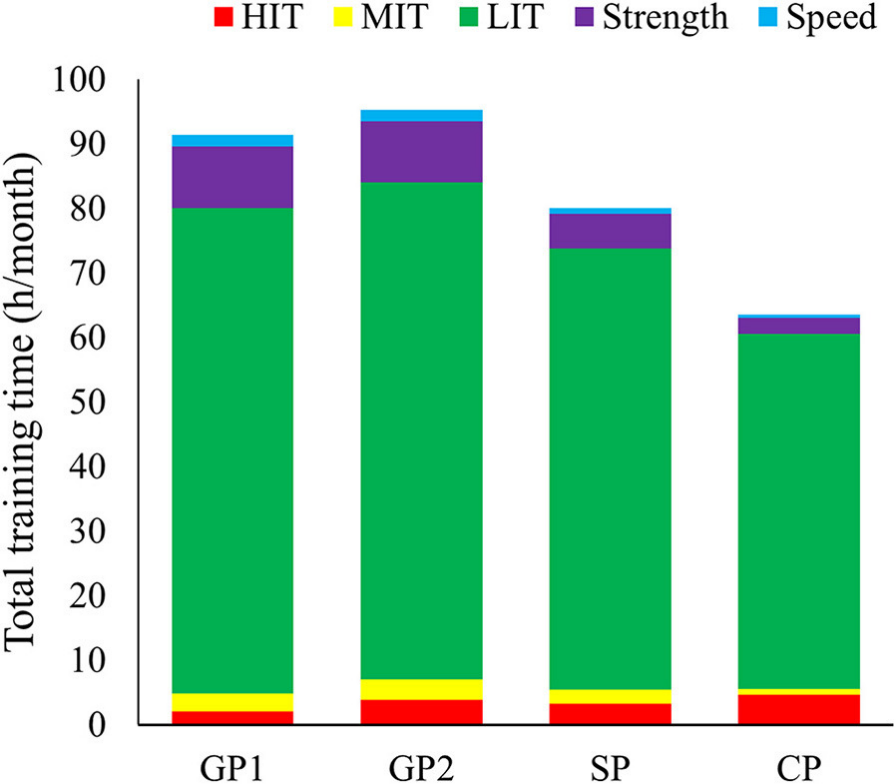
Training distribution across phases during the successful 2010–2015 period distributed into endurance (low-, moderate-, and high-intensity), strength and speed training for the world's most successful female cross-country skier.

A total of 2,713 training sessions, performed in the period from May 2010 to April 2015 were categorized based on the detailed design of the session (Table 3). Total annual training volume was 937 ± 25 h, distributed across 543 ± 9 sessions. This consisted of 849 ± 18 h (91%) endurance training, 75 ± 21 h (8%) strength training and 14 ± 2 h (1%) speed training. Monthly and weekly training patterns during different phases of the annual cycle are presented in Figure 2 and Table 4.

#### Endurance training

Using the modified session goal approach, 92.3 ± 0.3% of total endurance training time was executed as LIT, 2.9 ± 0.5% as MIT and 4.8 ± 0.5% as HIT (including competitions). When all endurance sessions were categorized using the session goal method, the distribution was 76.1 ± 1.1% LIT sessions, 7.3 ± 1.2% MIT sessions and 16.6 ± 1.2% HIT sessions.

Annual LIT volume was 784 ± 10 h. Monthly LIT volume decreased from GP (76 h), to SP (68 h) and further to CP (55 h) while the number of LIT sessions remained relatively stable across all phases (31.8 ± 1.6 sessions/month). Hence, the average duration of LIT sessions was reduced from GP (2.0 h) to SP (1.5 h) and CP (1.3 h). Total LIT time was distributed as 4% sessions <50 min, 10% as sessions [50–90> min, 42% as sessions [90–150> min and 23% as sessions ≥150 min. The remaining 21% of LIT time was performed as warm-up or cool down in connection with MIT, HIT, or strength sessions. The number of LIT sessions in the different categories during the different phases are presented in Figure 4A.

Annual MIT volume was 24.6 ± 3.6 h. Monthly MIT volume decreased from GP (2.8 h) to SP (2.2 h) and further to CP (1.0 h). The monthly number of MIT sessions was relative stable across GP1, GP2, and SP (3.5 sessions), but decreased markedly in CP (2 sessions). Average duration of MIT sessions decreased from GP (0.8 h) to SP (0.7 h) and CP (0.5 h). The annual number of 35 ± 5 MIT sessions consisted of 20% continuous sessions, 48% interval sessions with interval-durations from 6 to 10> min, and 22% as interval sessions with interval-durations from 10 to 15 min. The most common MIT session was an interval session consisting of 5 × 7–8 min working periods, with 1–2 min rest in between. The use of specific MIT sessions during different phases of the annual cycle is presented in Figure 4B.

Annual HIT volume was 40.4 ± 3.6 h. Monthly HIT volume increased from GP1 (2.0 h) to GP2 (3.7 h), was slightly reduced in SP (3.2 h) and then increased in CP (4.7 h). The monthly number of HIT sessions increased from GP1 (4.1 sessions) to GP2 (7.0 sessions), SP (8.2 sessions) and CP (9.2 sessions). Average duration of HIT sessions was approximately equal (0.5 h) across all phases except from SP (0.4 h). The number of annual competitions was 38.6 ± 6.3 (~70% distance- and ~30% sprint-competitions). Competition time increased from GP (0.5 h/month), to SP (1.6 h/month) and further to CP (3.1 h/month). Competitions accounted for 42 and 49% of total HIT time and number of HIT sessions, respectively. The annual number of 79 ± 8 HIT sessions consisted of 45% continuous sessions (including distance competitions), 38% interval training with interval-durations from 4 to 7 min and 14% intervals with interval-durations <4 min (including sprint competitions). The most typical HIT interval session was 5 × 4–5 min with 2–3 min rest in between. The use of specific HIT sessions during different phases of the annual cycle is presented in Figure 4C.

#### Strength and speed training

An important change during the five investigated years was an increase in annual strength training time from ~51 h (43% core stabilization and 57% heavy strength training) during the first 2 years (30–32 years old), to ~90 h (50% core stabilization and 50% heavy strength training) in the following 3 years. This increase was due to both an increased number (55–75 sessions) and duration (0.9–1.2 h) of sessions. The amount of strength training increased from 6.0 to 10.9 h/month in GP, from 2.6 to 4.2 h/month in SP and from 1.7 to 3.0 h/month in CP. The proportion of heavy versus core/stabilization training across phases was relatively similar during all 5 years, with the amount of heavy strength training increasing from GP (~50%), to SP (~60%) and further to CP (~65%).

A typical strength session consisted of 30–45 min of core/stabilization exercises followed by 30–45 min of heavy strength training. The core stabilization portion included various exercises targeting muscles involved in the force transfer during specific ski movements and exercises aiming to stabilize and move these segments functionally while skiing. Heavy strength sessions consisted of one or two leg exercises (e.g., squats) and three to four upper-body exercises (e.g., seated pull-down, standing double poling, pull-ups, lying bench-pull, and pullover).

Annually, 14 ± 2 h of speed training, including 11.1 h ski-specific exercises and 2.5 h of jumps/plyometrics, was performed. The amount of speed training decreased from GP (1.7 h/month) to SP (0.9 h/month) and CP (0.5 h/month). Speed training was included 64 ± 9 times/year and typically performed as 6–10 × 10–20 s sprints or 5–8 series of 10–15 plyometric jumps using ski specific movements integrated into LIT sessions of 90–120 minutes or performed before strength sessions.

#### Exercise modes

**Figure 3:**
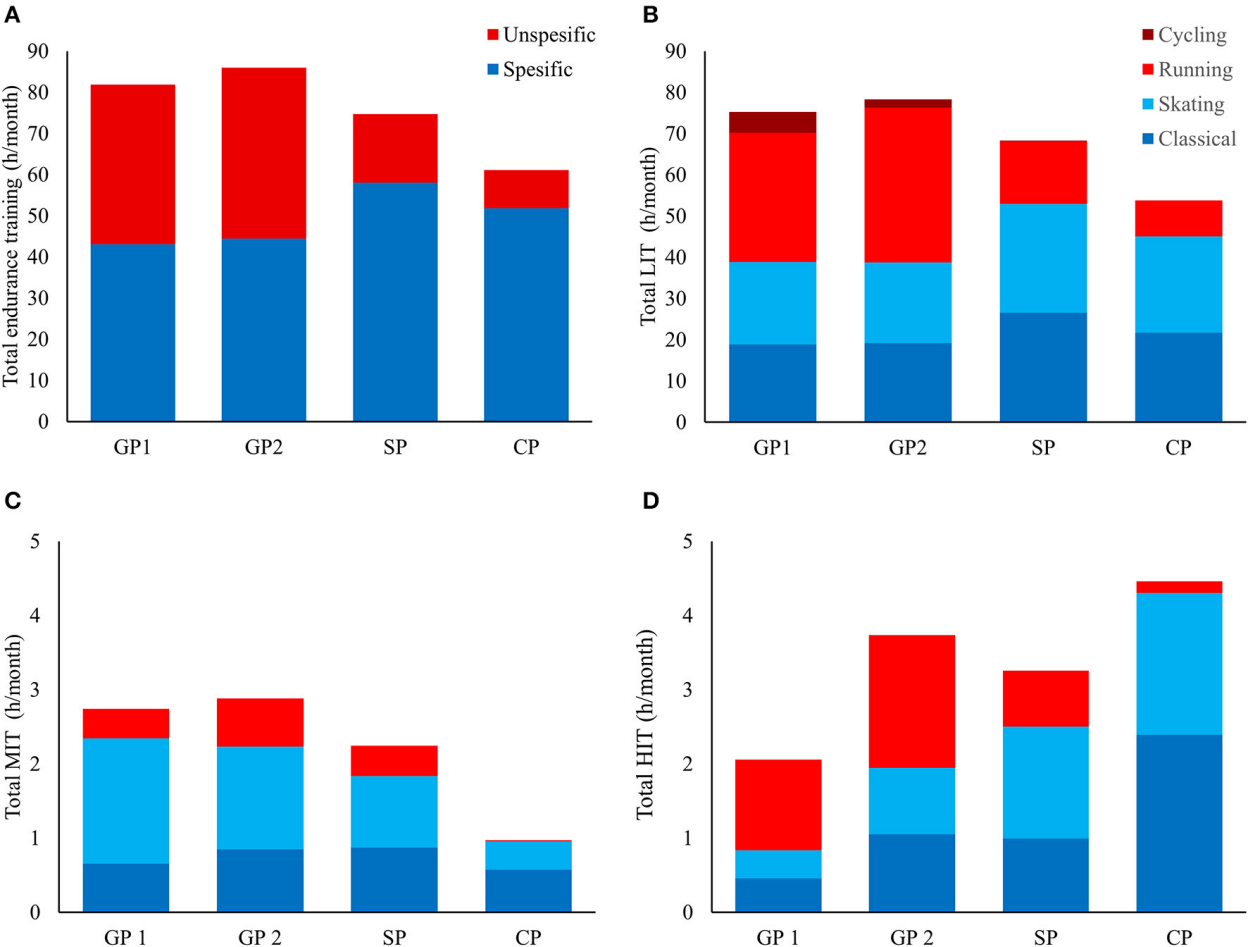
**(A–D)** Distribution of specific (skiing classical or skating) and non-specific activity forms (running and cycling) presented as total endurance and speed training time **(A)**, low—intensity training time **(B)**, moderate—intensity training time **(C)** and high—intensity training time **(D)** across phases during the successful 2010–2015 period.

63 ± 3% (545 ± 18 h) of the yearly endurance and sprint training was performed as sport-specific exercise modes (i.e., skating and classical on skis or roller skis), with the remaining 37 ± 2% (318 ± 18 h) performed as non-specific activity forms (34% running and 3% cycling). The proportion of specific activity forms increased from GP (52%) to SP (78%) and further to CP (85%). Specific training time also increased from GP (44 h/month) to SP (58 h/month), but then decreased slightly to CP (52 h/month). Sport-specific training accounted for 62, 83, and 72% of the annual LIT, MIT, and HIT volume, respectively. Figures 3A–D illustrate the distribution of activity forms across the different intensities and training phases. The distribution of training in the classic and skating techniques were approximately equal in total training time (48 and 52%), LIT (49 and 51%), and HIT (49 and 51%), while the proportion of skating was substantially higher during MIT (61%).

**Figure 4:**
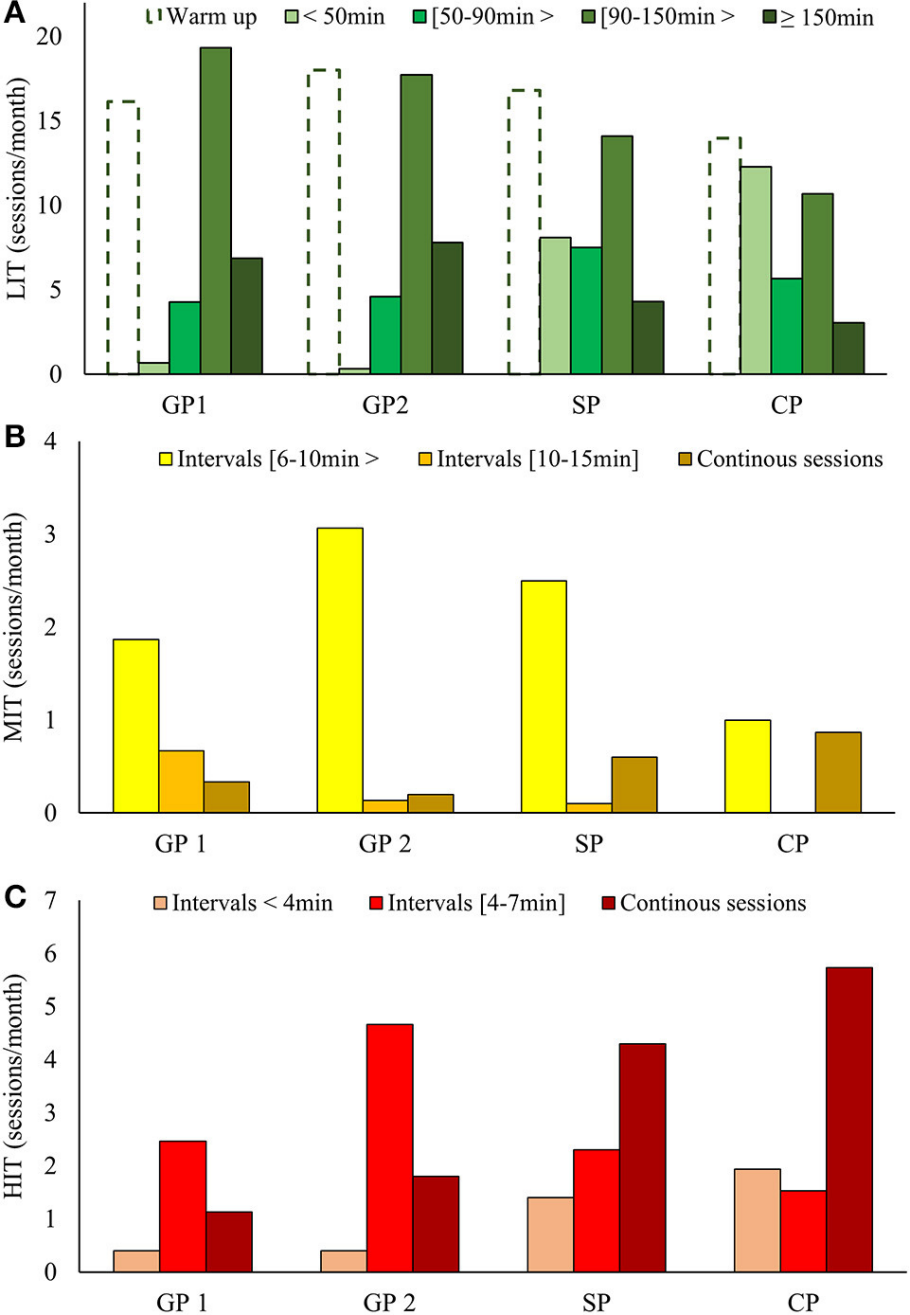
**(A–C)** Distribution of low- **(A)**, moderate- **(B)** and high-intensity **(C)** sessions, categorized after duration and/or organization, across phases during the five most successful years (2010–2015).

#### Altitude training

Total annual days spent at altitude was 61 ± 9, which were mainly distributed across five altitude camps (12–14 days June/July, 12–14 days August/September, 14–16 days October/November, 10–14 days in December and 10–12 days January/February). Total training volume at altitude ranged from 170 to 230 h, accounting for 18–25% of the total annual training volume. The average weekly training volume decreased from altitude camps performed in GP (~26 h) to SP (~22 h) and further to CP (~20 h).

**Figure 5:**
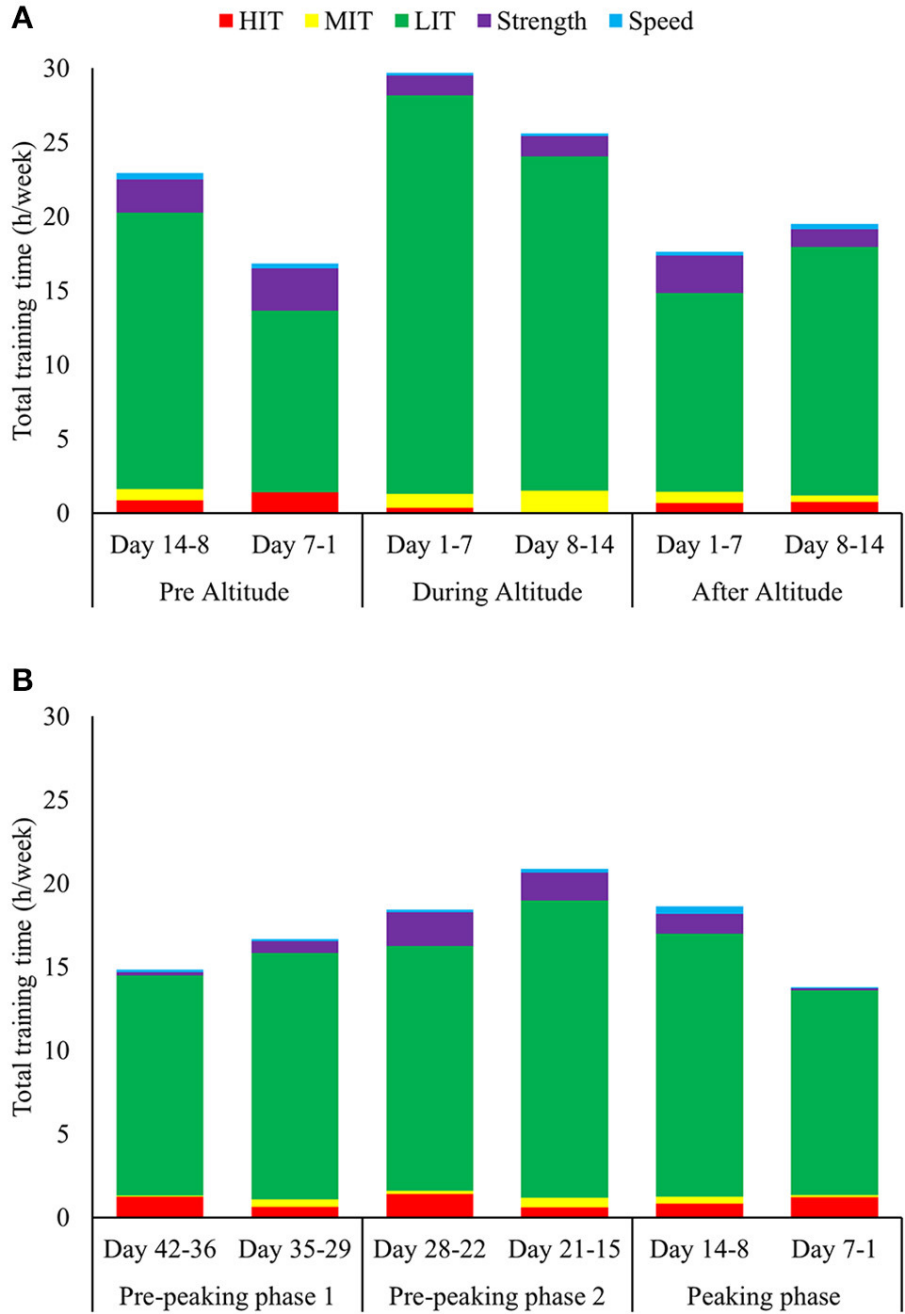
**(A,B)** Total weekly training distributions of **(A)** the 2 weeks before-, 2 weeks during- and 2 weeks after the annual altitude camp in October, and **(B)** the final 6 weeks before the four international championships performed in February during the period from 2010–2015, distributed into endurance training (low-, moderate-, and high-intensity), strength- and speed training.

Training during the 2 weeks before, 2 weeks during and the 2 weeks after the altitude camp in October/November are presented in Table 4 and Figure 5A. Total training volume was ~35% higher during altitude than the phases before and after. The increased training volume occurred due to an increased number of LIT session's ≥2.5 h, whereas strength training time was lower during altitude compared to the phases before and after. The amount of training in specific modes increased markedly at altitude, while the total volume of MIT and HIT remained stable (~1.5 h/week) across all three phases. However, the MIT/HIT distribution changed from containing more HIT before altitude (0.4 h MIT vs. 1.1 h HIT), but more MIT during altitude (1.2 h MIT vs. 0.2 h HIT) and equal amounts of MIT and HIT after altitude training (0.7 h MIT vs. 0.7 h HIT).

#### Tapering toward international championships

The distribution of training during the final 6 weeks prior to gold medal performance is presented in Table 4 and Figure 5B. Here, training volume increased by 25% from pre-peaking phase 1 (day 42–29) to pre-peaking phase 2 (day 28–15), due to increased amount of long duration LIT sessions and strength training. The training volume was then reduced by 18% to the peaking phase (day 14–1), which included a modest reduction in volume (6%) during the first week (day 14–8) and a larger reduction (30%) in the final week (day 7–1). The training volumes during the three consecutive peaking phases were 73, 91, and 75 % of the average GP2 training volume, respectively, while the number of sessions was stable across all phases (~11 sessions/week).

The amount of MIT+HIT volume remained relatively stable across all peaking phases (~1.3 h/week). Week-by-week analyses showed a progressive increase in the proportion of HIT vs. MIT during the final 3 weeks before the championship start. A detailed description of the specific sessions performed during the final 14 days before the 2014 Olympic Games is presented in Table 5.

**Table 5:** Detailed description of the training performed during the final 14 days before the 2014 Olympic Games in Sochi, including information about the commonalities during the same period before the World Championships in 2011, 2013, and 2015 for the world's most successful female cross-country skier.

	**Training content**	**Commonalities 2011, 2013, 2014, and 2015**
14	AM: 2.5 h LIT, ski skating on varying terrain PM: Warm-up + 30 min strength training^#^	**Day 14–8 before first championship event:** Second part of 10–12 days altitude camp at 1,800 m.a.s.l (i.e., the entire altitude camp was 8–20 days before the first competition) Training volume 17–20 h 2–3 LIT sessions >2.5 h 2 MIT/HIT sessions performed at 1,000 m.a.s.l 1–2 strength sessions 2–4 LIT sessions whit integrated sprints 1 rest day
13	Rest day	
12	AM: 2.5 h LIT, classical skiing on varying terrain PM: 1.3 h LIT, ski skating on varied terrain, including sprints	
11	AM: 5 × 7-min MIT^*^, ski skating on varied terrain PM: 1.3 h LIT, classical skiing on varied terrain, including sprints	
10	AM: 2.7 h LIT, classical skiing on varied terrain PM: Warm-up + 30 min strength training^#^	
9	AM: 2.3 h LIT, ski skating on varied terrain, including sprints PM: Rest	
8	AM: 6 min MIT + 5-km HIT^*^, classical skiing varying terrain PM: 0.5 h LIT, running	
7	AM: 1.3 h LIT, classical skiing on varied terrain, including sprints PM: 0.5 h LIT, running	**Day 7–1 before first championship event:** Training at championship elevation Total training volume of 13–16 h 3–4 HIT/MIT sessions Frequent medium and short duration LIT sessions Timing of sessions ▪ Day 6–4: 1–3 competitions ▪ Day 3: Easy day with LIT ▪ Day 2: HIT session or easy training with LIT ▪ Day 1: Easy training or short duration MIT session
6	MO: 0.5 h LIT, running AM: 10-km classic competition^*^ PM: 0.5 h LIT, running	
5	AM: Sprint skating competition^*^ PM: 0.5 h LIT, running	
4	Rest day with traveling	
3	AM: 1.3 h LIT, ski skating on varied terrain PM: 1.5 h LIT, classical skiing on varying terrain	
2	AM: 30 min HIT^*^, duathlon ski classical and skating varying terrain PM: 0.5 LIT, running	
1	AM: 1.3 h LIT, classical skiing on varying terrain PM: 0.5 h LIT, running	
0	Gold medal, skiathlon Olympic Winter Games Sochi 2014	

*LIT, low-intensity training**:** heart rate <87% max; MIT, moderate-intensity training**:** heart rate 87–92% max; HIT, high-intensity training**:** heart rate > 92% max*.

* *MIT and HIT sessions normally included 30–45 min of LIT as warm up and 15–30 min LIT as cool-down*.

# *Strength training sessions normally included 30–45 min of LIT as warm u*.

## Discussion

The present study investigated the training routines during the best-performing period of the most successful female XC skier of all time, analyzed in the context of her longitudinal training patterns. Following a 12-year progressive, non-linear increase in training load, the annual training volume during these 5 years was ~940 h, consisting of 91% endurance-, 8% strength-, and 1% speed training. Endurance training was gradually more polarized, due to reduced LIT and increased HIT, from GP to CP. 18–25% of the annual training time was done at altitude, performed with relatively short training camps (≤16 days) where HIT training is reduced and LIT training increased compared to sea-level training. Training before international championships included a 2-week increase in LIT and strength volume followed by gradual reduction in total training volume and increased HIT during the final week.

### Longitudinal and general training characteristics

In this study, in which 17 years of training were analyzed, our participant had a 12-year progressive, non-linear increase in training load from the age of 20 until the successful 5-year period analyzed in detail, where annual training volume was ~940 h, distributed across ~540 sessions. The overall progression in training mainly included an increase in LIT, although in a 6-year period she also increased the amount of HIT due to extensive blocks of HIT training during GP. The participant was already at a high international level and achieved her first international gold medal at 23 years old, with training volumes slightly in excess of 700 h. However, before the age of 25 she was not stable at the top level in distance races and mainly performed at a world-class level in sprint skiing; after which she performed equally well in all disciplines and techniques. While these data highlight the importance of a high training volume to achieve a top international level in XC-skiing (Tonnessen et al., 2014; Sandbakk et al., 2016; Sandbakk and Holmberg, 2017), they also indicate that a progressive increase in training load is beneficial.

Throughout the 12 initial years with increases in training load, our participant had two major changes in “training philosophy,” in which rapid performance improvements also occurred. The first of these periods was in an early stage of her senior career, where extensive blocks of HIT were included. This led to rapid performance improvements that stagnated after a few years, during which the progression in training load and/or variation in stimulus were limited. The next major performance improvement coincided with a change to a more even distribution of training volume/intensity, a reduction in the amount of HIT, and the implementation of relatively large amounts of LIT. This occurred directly before entering the successful 5-year period analyzed here. In this period, both physiological values and performance improved over the first year before remaining at a stable high level. The annual training volume of >910 h consisted of ~850 h endurance training distributed as 92% LIT, 3% MIT, and 5% HIT when using the modified session-goal approach to quantify training. When quantified by the number of sessions in each zone, ~475 endurance sessions were distributed into 76% LIT, 7% MIT, and 17% HIT. While these amounts of HIT and MIT are similar to what was previously reported in world-class XC skiers (Sandbakk et al., 2011b, 2016; Tonnessen et al., 2014), the volume of LIT is remarkably high. This was combined with relatively high amounts of strength training and regular speed training, which may have been beneficial for maintaining muscle mass and sprint ability. A further progression in stimuli was achieved through inclusion of more strength training halfway through the 5-year period, while the amount of endurance training remained relatively stable. One unique feature of our participant is that she combines a high aerobic capacity with greater muscle mass than normally reported among female XC skiers (Hegge et al., 2016), particularly in the upper-body where women typically have the largest difference in body composition and performance compared to men (Sandbakk et al., 2017).

In XC skiing, not just exercise volume, frequency, and intensity are of importance. Through the 17-year period, ~60% of annual endurance and sprint training hours were ski specific (skiing on snow or roller skis), while the rest was primarily running. This alternation between exercise modes, loading the whole body, upper body, and the legs to a different extent, is unique for XC-skiing compared to other endurance sports. The variation between employment of these training modes permits high training loads during GP, while the total training load is reduced when less variation and increased sport-specific training is used toward CP. However, variations between exercise modes were also employed on the micro periodization level; e.g., by performing heavy strength training of the upper body in the morning session followed by lower body endurance training (e.g., running) in the afternoon. This way of loading the upper and lower body may not only increase the tolerable training load, but could also reduce negative cross-over adaptation effects from concurrent strength and endurance training. In our case, the participant confirmed during interviews that she was conscious about the use of terrain, e.g., by combining uphill sessions where the legs are mainly employed, with sessions primarily loading the upper body by using the double poling technique on the same day. This is likely an important factor contributing to the combination of high endurance capacity and a relatively large muscle mass obtained by XC skiers.

Following a gradual increase in aerobic capacity, the participant's average *V*O_2max_ was ~68 (ml·kg ^−1^·min^−1^) during her five most successful years. This is at the same level reported in female champions in running and orienteering (Jones, 2006; Tonnessen et al., 2015b). Her AT increased correspondingly, and both the participant and her coaches highlighted her gradually improved ability to train with relatively high speed and a high technical quality also during LIT and MIT sessions in all exercise modes. This is supported by her lactate profiles, where her speed at various submaximal lactate levels gradually increased throughout her career. Similar results were shown in the female marathon world record holder (Jones, 2006) and a world-class rower (Bourgois et al., 2014). This is most likely a result of her long-term progressive increase in endurance training load, leading to enhanced peak oxygen uptake, fat utilization and improved efficiency in all exercise modes. In this context, it is also important to note that the body mass of the participant was very stable throughout her senior career, and measurements of body composition during the five successful years showed that both her fat percentage and bone mineral density were within healthy values. We suggest that this is an important reason for her continuity in training during the 17 years with high loads of endurance training.

Overall, our data indicate that a progressive increase in training load until the age of 30 may be necessary in order to optimize the full potential of a top-level XC skier. We hypothesize that this allowed our champion XC skier to tolerate and respond positively to the high training volumes utilized in the 5-year period analyzed, where she used a polarized training pattern with a large amount of LIT.

### Training characteristics during five successful years

#### Annual periodization of training

The total number of LIT sessions remained stable across phases throughout the training year, while total LIT-time was gradually reduced from GP to CP. The amount of MIT, speed, and strength training also decreased from GP to CP, while HIT showed the opposite pattern, which altogether induced a gradually more polarized training pattern toward CP. The transition from a more “pyramidal” to a more polarized endurance training pattern was previously shown in successful athletes (Stoggl and Sperlich, 2015). However, the large amounts of speed and strength training during GP might be an important addition to concurrently develop endurance and strength capacities during the preparation period, whereas the subsequently more polarized pattern may facilitate the ability to utilize these capacities at competition-specific intensities.

Simultaneously, the amount of specific training increased from 50% during GP to 85% in CP. This is in line with previous studies of XC skiers and probably functions as an important substitute for reduced volume during CP (Tonnessen et al., 2014). While the sport-specific proportion of LIT and HIT increased markedly from GP to CP, the amount of specific MIT was >80% during all phases. In addition, the MIT sessions were performed at relative high heart rates (87–92% of HR_max_), which is higher than normally reported in elite athletes, although RPE ratings and lactate values were in the normal range for such sessions. The participant confirmed that she was able to perform MIT sessions at this level, which allowed her to accumulate and tolerate much more time at >90% of HR_max_ than most of her peers. Such training has previously been reported to be highly effective for endurance adaptations and performance in well trained elite athletes (Stepto et al., 1999; Sandbakk et al., 2013).

The accumulated LIT-time during GP was very high (76 h/month) and reduced in CP (55 h/month). While the number of LIT sessions remained stable across phases (~32 sessions/month), the amount of LIT sessions ≥90 min decreased from GP (25 sessions/month) to CP (14 sessions/month). Another pronounced change between phases was the increase of LIT sessions <50 min from GP (~0 sessions/month) to CP (~14 sessions/month). This methodological approach is novel, and clearly shows how LIT sessions of different duration are distributed differently throughout the year. The effect of duration versus frequency of LIT sessions has not yet been examined, although up to 90% of the total training among endurance athletes is LIT. Interestingly, 21% (167 h) of the annual LIT volume was warm up or cool down in connection with MIT, HIT, or strength sessions. This part of LIT probably functions as an important contributor to the long-term development by enhancing the total training volume.

While the majority of MIT and HIT sessions were organized as intervals during GP, an increase in continuous MIT and HIT sessions was observed as the CP approached. Both exercise mode, organization of HIT, and use of terrain got more specific closer to CP. The fact that 42% of annual HIT time was competitions emphasizes the importance of specific training to achieve success in XC skiing. The participant also confirmed that competitions were an important part of her training, particularly during her tapering phase. The organization of endurance sessions changed from longer to gradually prioritizing shorter LIT sessions, while MIT and HIT sessions became more competition-specific.

#### Altitude training

18–25% of annual training volume was performed during relatively short (10–16 days) altitude camps, living at 1,800–2,000 m.a.s.l and training at 1,000–3,000 m.a.s.l., with a clear reduction in HIT but an increased volume of LIT compared to sea-level training. This altitude exposure is significantly shorter than the 4 weeks recommended to fully stimulate erythropoiesis. However, comparable duration of camps is reported to have beneficial effects on work economy, muscle buffering capacity, and ventilatory factors (Millet et al., 2010). Furthermore, the long-term effect of repeated short-duration altitude exposure over several years is currently unknown.

The participant experienced marked progress after altitude training, although it is not known to what extent altitude-facilitated effects and/or the periodization of training occurring in connection with altitude camps influence the experienced progress. Before altitude, training changed toward a more polarized pattern, including lower total volume and more HIT and strength training. During altitude, HIT was reduced and training shifted to a more pyramidal intensity distribution, with more LIT sessions ≥2.5 h and increased amounts of MIT. Training after altitude consisted of some easy days with reduced volume and no MIT or HIT during the first 4 days after altitude exposure, followed by increased intensity in training. The participant also highlighted the opportunity to ski on snow, more time to rest, and an increased focus on recovery as possible factors contributing to the positive effect of altitude camps.

#### Tapering toward international championships

The tapering phase prior to international championships included a phase with frequent competitions, followed by elevated training volume including more LIT and strength at altitude. Thereafter, our participant reduced her training volume and increased the amount of HIT during the final week before championships. However, the reduction in training volume during the final 2 weeks (18%) was much lower than recommendations in the literature (Bosquet et al., 2007). The same observation was made by Tonnessen et al. (2014), where the authors speculated that this might be optimal in sports with a dense competition schedule. As such, top athletes in XC skiing appear to reduce their training volume less than that recommended by the current literature. Maintenance of training volume until the final week before the first championship event could also be important in order to maintain performance level over 5–6 competitions during a championship lasting 9–15 days.

Our participant integrated the competition schedule into the tapering strategy and had a relatively similar timing of the final competitions in the peaking phase during all 5 years. Specifically, a period with frequent competitions, allowing less training hours, is followed by a competition break, prioritizing altitude training with more MIT, long duration LIT sessions and strength training. Thereafter, three HIT sessions were performed during the final 7 days, which include competitions at day 6–4 before the championship's start. However, since this analysis is based on the training conducted prior to the first competition in each championship, it is not certain that this was the day with the best performance (although gold medals were won already at the first competition).

## Conclusion

Our study supports previous findings highlighting the importance of a high training volume, using a polarized training pattern with a large amount of LIT to reach world-class level in XC skiing. This study provides unique data on the world's most successful XC skier's long-term training process, including novel information about the physiological development and the distribution of and interplay between sessions of different training forms, intensities, and exercise modes throughout the annual season. By using a single-case approach, where quantitative data were supported by qualitative interviews, we were able to present the sophisticated training of a world-class athlete from a macro- to a micro-level, allowing the generation of new hypotheses that can be tested in future research with larger samples.

## Author contributions

GS, ET, and ØS designed the study; GS performed data collection; GS, ET, and ØS performed data and statistical- analysis; GS, ET, and ØS contributed to interpretation of the results; GS and ØS wrote the draft manuscript; GS, ET, and ØS contributed to the final manuscript.

### Conflict of interest statement

The authors declare that the research was conducted in the absence of any commercial or financial relationships that could be construed as a potential conflict of interest.
